# Case-Control Study Design to Identify Attributable Risk Factors for Rare Yet Trending Healthcare-Associated Infections

**DOI:** 10.1017/ash.2024.303

**Published:** 2024-09-16

**Authors:** Stephanie Stroever, Krystal Hill, Charity Conley, Teri Quattlebaum

**Affiliations:** Texas Tech University Health Sciences Center; Northwest Texas Healthcare System

## Abstract

**Introduction:** There are a number of tools available to healthcare epidemiologists for identification and investigation of healthcare-associated infections. For example, root cause analysis (RCA) is an evidence-based strategy for identifying process failures that resulted in infection. However, RCA is most valuable on a case-by-case basis. It is not an efficient tool for investigating numerous events that trend over time. Healthcare epidemiologists must use different strategies that are both efficient and powerful. A case-control study is a valuable option to investigate rare but recurrent infection events. The objective of this study was to demonstrate the utility of a case-control study design to detect attributable risk factors of cesarean section surgical site infections (SSI). **Methods:** We conducted a case-control study at a Level III childbirth center with data timeframe of January 1, 2021 to May 31, 2023. The project was approved by the institutions’ Quality Improvement Review Board prior to implementation. Cases were identified using the National Healthcare Safety Network (NHSN) SSI event criteria and included all levels of SSI. Controls were selected from the NHSN surgical denominator and were matched randomly without replacement by age at a 1:4 ratio. Variables were identified in collaboration with stakeholders based on known risk factors for SSI and abstracted manually. Analyses were conducted in Stata/MP version 17.0, and we performed multivariable conditional logistic regression with an alpha of 0.05 as threshold for statistical significance. The model was built according to a priori hypotheses and results from bivariate analysis of individual risk factors. **Results:** There were 32 SSI among 1709 cesarean deliveries, and all cases successfully matched with 4 controls for an analytic sample of 128. Bivariate analyses identified 7 relevant variables for inclusion in the multivariable model which narrowed down significant risk factors to 3: operative time (in minutes), post-operative chlorhexidine gluconate (CHG) bathing, and number of people in the operating room. Assessment of fit indices suggested an excellent fit with pseudo R-square of 0.526. **Conclusions:** This study demonstrated the utility of a case-control study design to identify attributable risk factors for relatively rare but significantly trending infection events. Not only is the design more efficient (e.g., time needed to abstract 50 data points for each patient), but it also employs statistical analyses that are often lacking in case-by-case investigations or RCA. It also has the power to narrow down risk factors for focused prevention efforts to get “the most bang for the buck.”

